# The potential pathogenic roles of S100A8/A9 and S100A12 in patients with MPO-ANCA-positive vasculitis

**DOI:** 10.1186/s12865-022-00513-4

**Published:** 2022-09-10

**Authors:** Xue Bai, Peng-Cheng Xu, Tong Chen, Hao-Miao Zhang, Si-Jing Wu, Xia Yang, Shan Gao, Jun-Ya Jia, Jian-Qing Jiang, Tie-Kun Yan

**Affiliations:** 1 Department of Nephrology, Tianjin Medical University General Hospital, No. 154 Anshan Road, Heping District, Tianjin, 300052 People’s Republic of China; 2 Department of Hematology, Tianjin Medical University General Hospital, No. 154 Anshan Road, Heping District, Tianjin, People’s Republic of China

**Keywords:** S100A8/A9, S100A12, MPO, ANCA, AAV

## Abstract

**Background:**

The significance of S100A8/A9 and S100A12 in anti-neutrophil cytoplasmic antibody (ANCA)-associated vasculitis (AAV) has not been clarified. This study was dedicated to exploring the potential pathogenic roles of S100A8/A9 and S100A12 in patients with myeloperoxidase (MPO)-ANCA-positive vasculitis.

**Methods:**

Serum and urine concentrations of S100A8/A9 and S100A12 of forty-two AAV patients were evaluated. The influence of S100A8/A9 and S100A12 on the chemotaxis, the apoptosis, the release of IL-1β, the complement activation, the respiratory burst, as well as the neutrophil extracellular traps (NETs) formation of MPO-ANCA-activated neutrophils was investigated.

**Results:**

The serum and urine S100A8/A9 and S100A12 of active MPO-AAV significantly increased (compared with inactive AAV and healthy controls, *p* < 0.001) and were correlated with the severity of the disease. In vitro study showed that S100A8/A9 and S100A12 activated the p38 MAPK/NF-κB p65 pathway, increased the chemotaxis index (CI) and the release of IL-1β, extended the life span, and enhanced the complement activation ability of MPO-ANCA-activated neutrophils. The Blockade of TLR4 and RAGE inhibited the effects of S100A8/A9 and S100A12. All above-mentioned effects of S100A8/A9 and S100A12 were ROS-independent because neither S100A8/A9 nor S100A12 enhanced the ROS formation and NETs formation of MPO-ANCA-activated neutrophils.

**Conclusion:**

S100A8/A9 and S100A12 serve as markers for assessing the disease severity, and they may also play a role in MPO-AAV pathogenesis.

**Supplementary Information:**

The online version contains supplementary material available at 10.1186/s12865-022-00513-4.

## Background

Antineutrophil cytoplasmic antibody (ANCA)-associated vasculitis (AAV) is an autoimmune disease characterized by serum-positive ANCA which mainly recognizes myeloperoxidase (MPO) or proteinase 3 (PR3) and the rapidly progressive glomerulonephritis which shows pauci-immune complex deposition in pathogenic biopsy [1]. AAV encompasses microscopic polyangiitis (MPA), granulomatosis with polyangiitis (GPA), and eosinophilic granulomatosis with polyangiitis (EGPA) [2]. In AAV, the ANCA-activated neutrophils (polymorphonuclear lymphocytes, PMNs) extrude neutrophil extracellular traps (NETs), which are decorated by histones, MPO, PR3, neutrophil elastase (NE), as well as other cytoplasmic proteins [3].

Previous studies demonstrated that activated neutrophils could also extrude damage-associated molecular pattern (DAMP) proteins such as high mobility group box chromosomal protein 1 (HMGB-1) and some S100 family proteins [4]. HMGB-1 has been reported to take part in the pathogenesis of AAV [5, 6]. However, the role of the S100 family proteins in AAV has not been clarified. S100A8/A9 and S100A12 belong to the S100 protein family that was first extracted from cow brain by Blake W. Moore and his colleagues in 1965 [7]. Literature reported that S100A8/A9 could stimulate renal mesangial cells to release IL-6, TNF-α, and CXCL1 [8], while S100A12 enhanced cytokine expression in a dose-dependent manner and promoted the secretion of chemokines and cell adhesion molecules in the normal bronchial epithelial cells [9]. It has been reported that the serum levels of S100A8/A9 and S100A12 in AAV were elevated [10–12]. However, the exact pathogenic functions of S100A8/A9 and S100A12 in AAV with MPO-ANCA have no further refining study. In the current study, we tried to investigate the role of S100A8/A9 and S100A12 in MPO-ANCA-positive vasculitis.

## Results

### The serum and urinary levels of S100A8/A9 and S100A12 are associated with the clinical parameters of patients with active MPO-ANCA-positive vasculitis

**Fig. 1:**
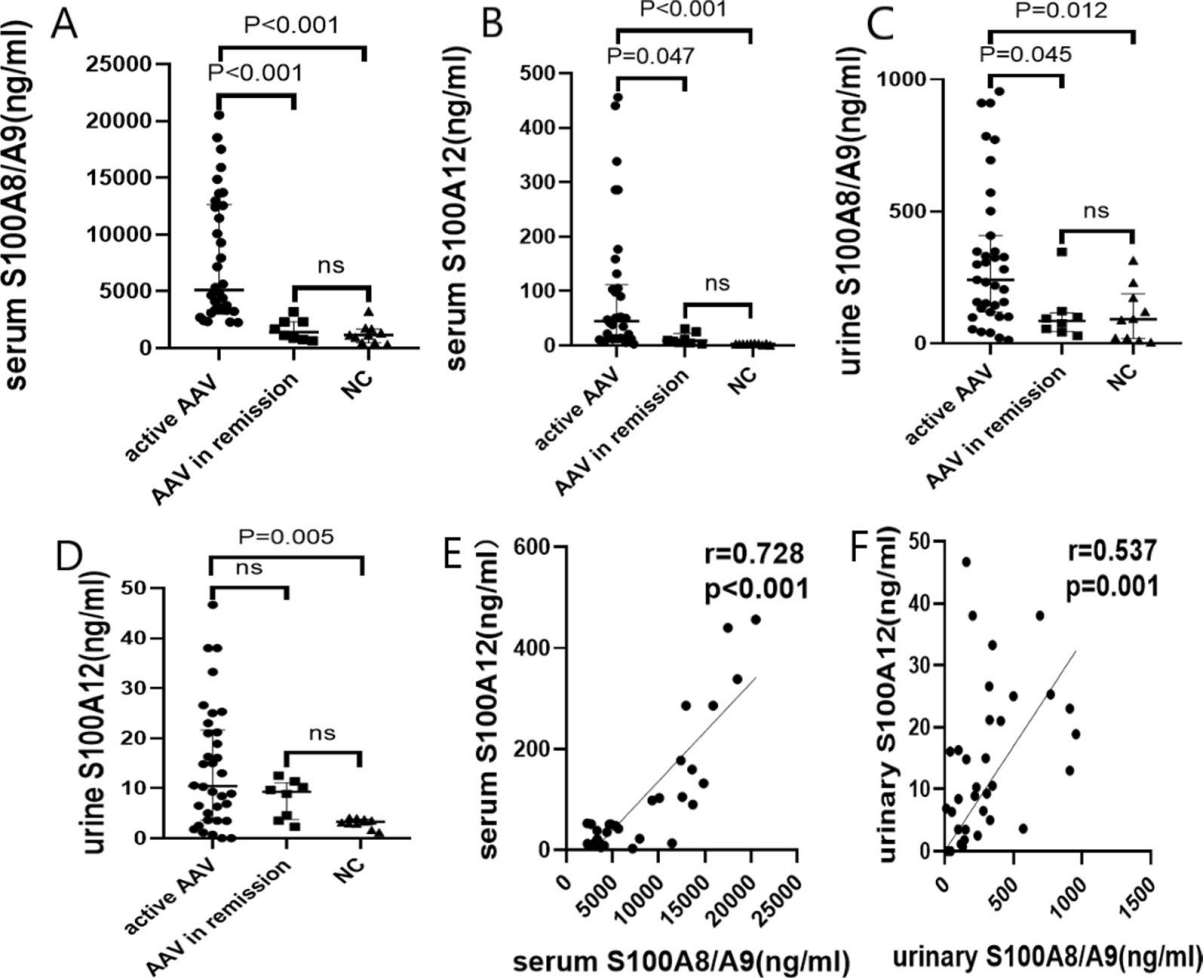
Levels of serum and urinary S100A8/A9 and S100A12 in MPO-AAV patients and normal controls. Comparison of concentrations of serum S100A8/A9 (**A**), serum S100A12 (**B**), urinary S100A8/A9 (**C**), and urinary S100A12 (**D**) between MPO-AAV patients in the active period or remission and NC. **E** The relationship between serum S100A8/A9 and serum S100A12 in active MPO-AAV. **F** The relationship between urinary S100A8/A9 and urinary S100A12 in active MPO-AAV. NC: normal controls. ns: not significant

Table 1 shows patients' clinical and laboratory data. Serum and urine from 42 AAV patients and ten healthy controls were tested for the levels of S100A8/A9 and S100A12. Serum levels of S100A8/A9 and S100A12 in active AAV were significantly higher than that in remission and normal controls (Fig. 1A and B). Patients in remission have no difference in their serum levels of S100A8/A9 and S100A12 compared with healthy controls (Fig. 1A and B). Compared with normal controls, the urinary S100A8/A9 and S100A12 of active patients were much higher (Fig. 1C and D). The urinary S100A8/A9 level, not S100A12, was raised in patients with active diseases compared with patients in remission (Fig. 1C and D). Figure 1E revealed that the concentration of serum S100A8/A9 was positively correlated with the serum level of S100A12 in 34 patients with active diseases. There was also a moderate relationship between urinary S100A8/A9 and S100A12 (Fig. 1F).

**Table 1:** The clinical and laboratory data of MPO-ANCA patients and healthy controls

Parameters	Active patients (n = 34)	Remission patients (n = 8)	Healthy controls (n = 10)	*P* value
Male/female	15/19	4/4	6/4	0.672
Age (years)	63 (57.75, 68.25)	64.5 (61, 67.50)	59.5 (37.25, 69.25)	0.577
BVAS	15 (13, 16.25)	0 (0, 0)		< 0.001
Scr (μmol/l)	182.5 (107.25, 286.75)	63 (48.25, 83.5)		< 0.001
HB (g/l)	97.82 ± 21.53	115.8 ± 17.24		0.035
WBC (10^9^/l)	8.55 ± 3.46	8.97 ± 3.49		0.757
PLT (10^9^/l)	275.18 ± 100.36	307.88 ± 153.25		0.460
ANCA levels (IU/ml)	154.89 ± 84.84	35.99 ± 21.69		< 0.001
CRP (mg/dl)	1.01 (0.27,6.91)	0.49 (0.39, 0.89)		0.364
C3 (mg/dl)	88.28 ± 17.99	96.98 ± 19.67		0.235
C4 (mg/dl)	25.85 ± 8.90	22.81 ± 8.27		0.386
ESR (mm/1 h)	55 (36, 65.5)	24.5 (14, 65.50)		0.015
SF (ng/ml)	333.46 ± 265.27	210.8 ± 160.8		0.210
D-Dimer (ng/ml)	1856 (652, 3935)	910 (582, 3938)		0.428
RF (IU/ml)	20 (20, 48.1)	30.95 (20, 49.88)		0.588
ALB (g/l)	31.88 ± 5.99	34.25 ± 5.50		0.314
Proteinuria (g/24 h)	1.55 (0.55, 1,99)			
Hematuria(number/μl)	224.5 (31.58, 712.08)			
NGAL (ng/ml)	32.68 (13.92, 74.26)			
Disease duration (months)	3 (1.43, 6.13)			
Renal	100%			
Lung	41%			
ENT	6%			
Joint	14%			
Gastrointestinal	9%			
Skin	12%			

BVAS: Birmingham vasculitis activity score; Scr: serum creatinine; HB: hemoglobin; WBC: white blood cell; PLT: platelet; ANCA: antineutrophil cytoplasmic antibody; CRP: C reactive protein; C3: complement 3; C4: complement 4; ESR: erythrocyte sedimentation rate; SF: serum ferritin; RF: rheumatoid factor; ALB: albumin; NGAL: neutrophil gelatinase-associated lipocalin; ENT: ear, nose, and throat

**Fig. 2:**
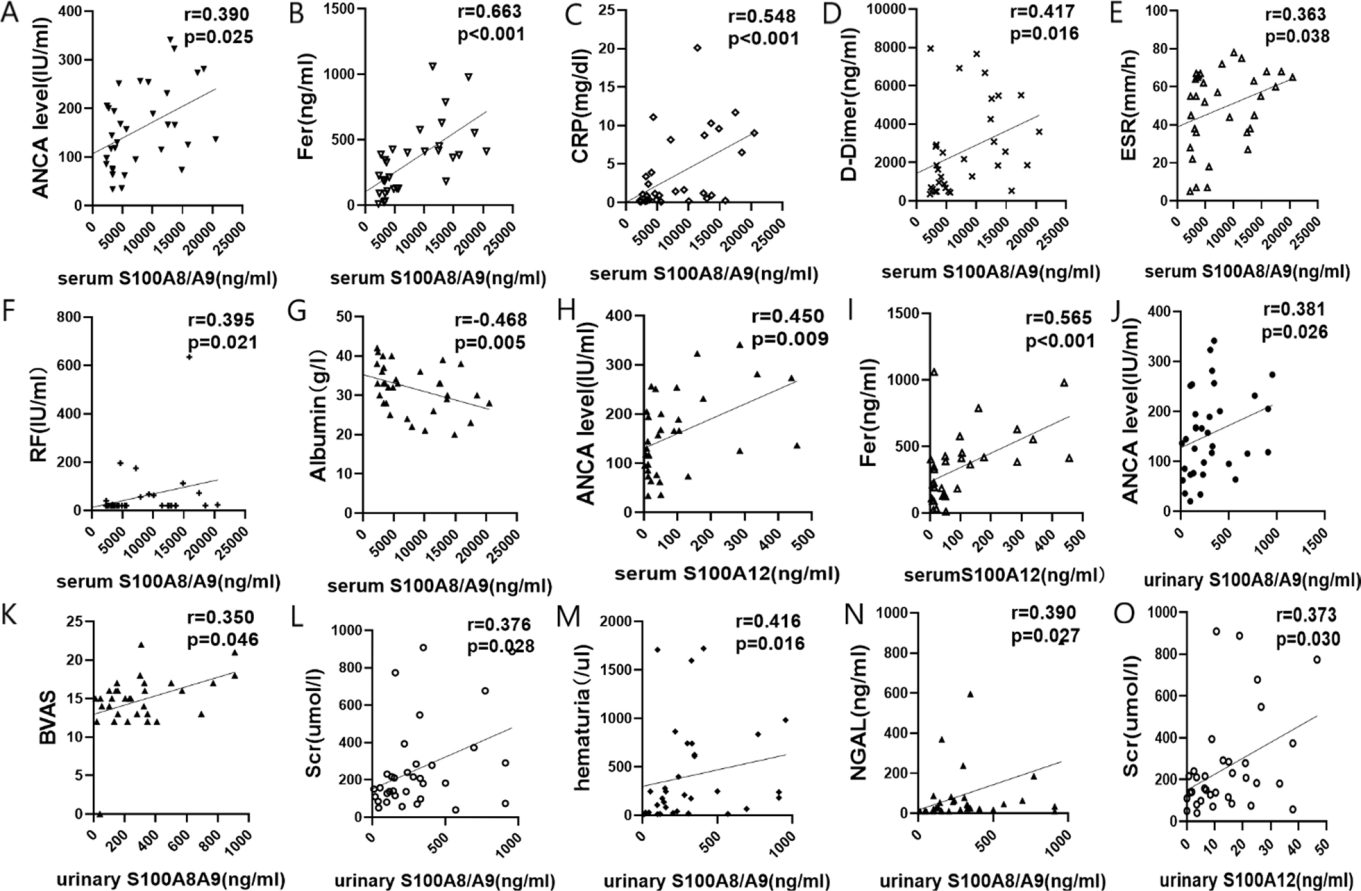
The correlations between serum/urinary S100A8/A9 and S100A12 and clinical parameters in 34 active MPO-AAV. **A**–**G** showed the correlations of serum S100A8/A9 and the level of MPO-ANCA (**A**), serum ferritin (**B**), CRP (**C**), D-Dimer (**D**), erythrocyte sedimentation rate (**E**), rheumatoid factor (**F**), serum albumin (**G**). **H** and **I** showed the correlations of serum S100A12 and the level of MPO-ANCA (**H**) and serum ferritin (**I**). **J**–**N** showed the correlations of urinary S100A8/A9 and MPO-ANCA (**J**), BVAS (**K**), serum creatinine (**L**), hematuria (**M**), and urinary NGAL (**N**). **O** Showed the correlation between urinary S100A12 and serum creatinine. BVAS: Birmingham vasculitis activity score. NGAL: neutrophil gelatinase-associated lipocalin

For 34 patients with active AAV, the serum concentration of S100A8/A9 was positively correlated with the level of MPO-ANCA, serum ferritin, C-reaction protein (CRP), D-dimer, erythrocyte sedimentation rate (ESR), and rheumatoid factor (RF) (Fig. 2A–F), negatively correlated with serum albumin (Fig. 2G), and had no correlation with Birmingham vasculitis activity score (BVAS) (r = 0.215, *p* = 0.229). The serum S100A12 level was closely correlated with the MPO-ANCA level and serum ferritin (Fig. 2H and I). However, there was no correlation between serum S100A12 and CRP, serum albumin, D-Dimer, RF, ESR, and BVAS.

The correlation analysis also showed that the urinary concentration of S100A8/A9 was correlated with the level of MPO-ANCA, BVAS, serum creatinine, hematuria, and the level of urinary neutrophil gelatinase-associated lipocalin (NGAL) of those 34 patients with active diseases (Fig. 2J–N). The urinary concentration of S100A12 was only correlated with serum creatinine (Fig. 2O) and had no correlation with the level of MPO-ANCA, BVAS, hematuria, or urinary NGAL (data not shown).

### ANCA stimulates neutrophils to release S100A8/A9 and S100A12 in vitro

**Fig. 3:**
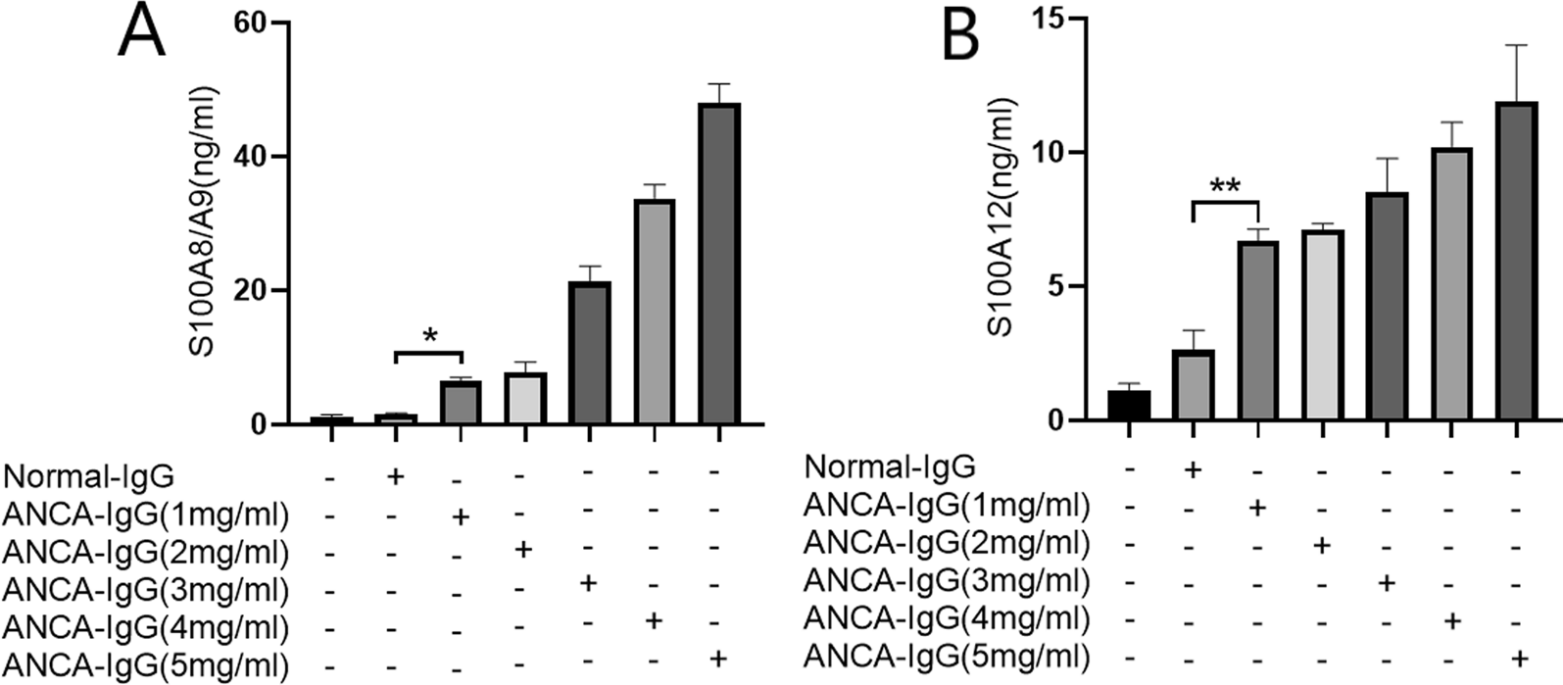
ANCA IgG stimulated neutrophils to release S100A8/A9 (**A**) and S100A12 (**B**) concentration-dependently. *p < 0.05, **p < 0.01

Since the serum concentrations of both S100A8/A9 and S100A12 increased in the patients with active MPO-AAV and were significantly related to the MPO-ANCA levels, we tried to investigate whether the ANCA-activated neutrophils could release S100A8/A9 and S100A12. We incubated neutrophils with MPO-ANCA-containing IgG. The supernatant S100A8/A9 significantly increased after neutrophils were incubated with 1 mg/ml ANCA-containing IgG (compared with the group of normal IgG, 6.47 ± 0.55 ng/ml vs. 1.56 ± 0.19 ng/ml, *p* = 0.011). Meanwhile, the S100A12 concentration was also elevated after neutrophils were incubated with MPO-ANCA-containing IgG (compared with the control of normal IgG, 6.70 ± 0.43 ng/ml vs. 2.60 ± 0.76 ng/ml, *p* = 0.004). Along with the concentration of ANCA-containing IgG increased, the S100A8/A9 and S100A12 secreted by neutrophils were also enhanced (Fig. 3A and B), which proved that MPO-ANCA could stimulate the release of S100A8/A9 and S100A12 in a concentration-dependent manner.

### MPO-ANCA enhances the expression of toll-like receptor 4 (TLR4) and receptor for advanced glycosylation endproducts (RAGE) on neutrophils

To evaluate the effect of MPO-ANCA on the expression of TLR4 and RAGE on neutrophils, we detected the proportion of TLR4/RAGE double-positive neutrophils. The proportion of TLR4^+^ RAGE^+^ neutrophils was significantly increased after neutrophils were treated with ANCA-IgG (Additional file 2: Figure S1F). Moreover, S100A8/A9 and S100A12 had the similar effects (Additional file 2: Figure S1F).

### S100A8/A9 and S100A12 induce chemotaxis and migration of neutrophils

**Fig. 4:**
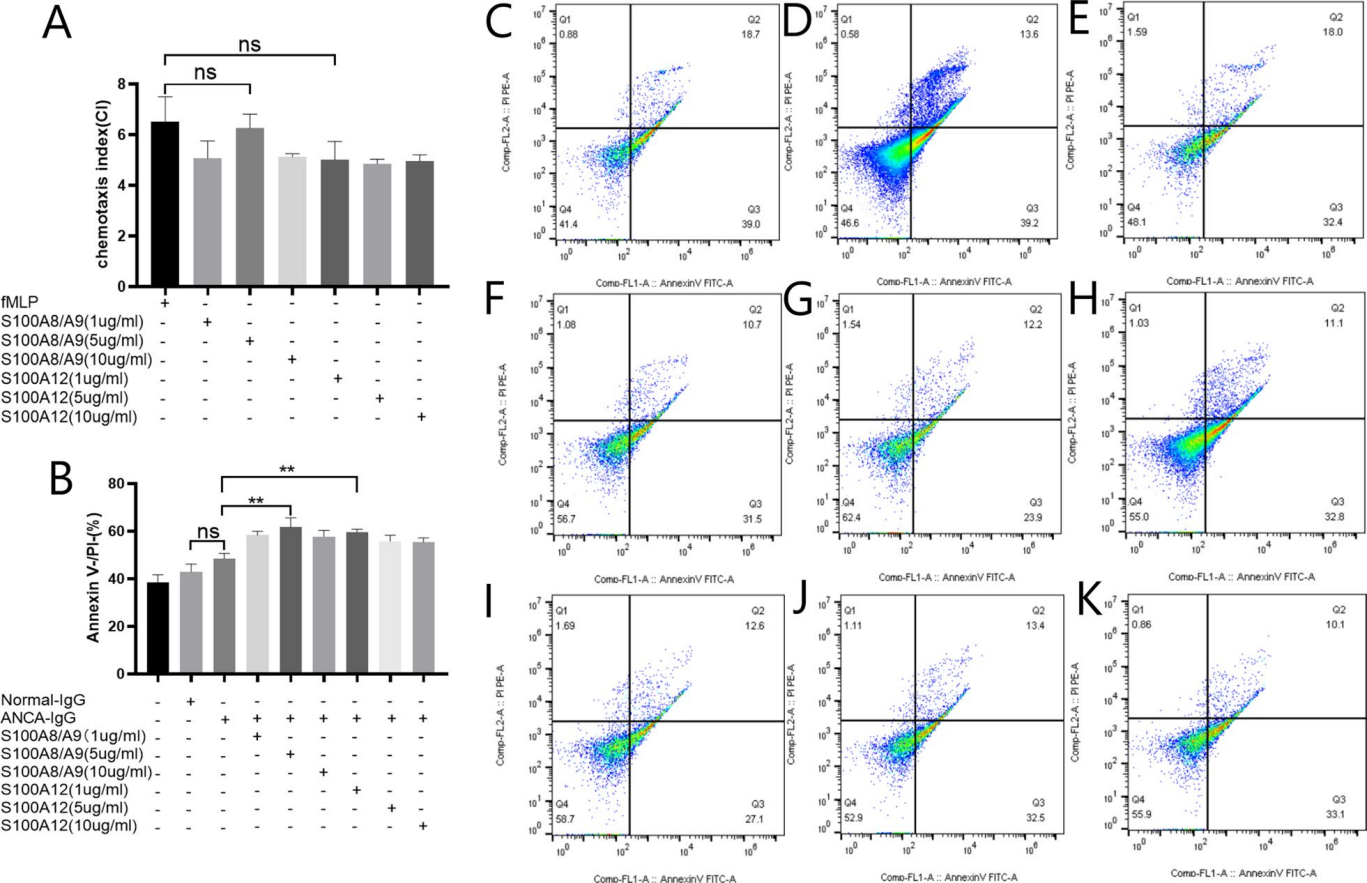
Influence of different concentrations of S100A8/A9, and S100A12 on the chemotaxis and cell death of neutrophils. **A** The chemotaxis index of ANCA-activated neutrophils treated with different concentrations of S100A8/A9 and S100A12. **B** Effects of S100A8/A9 and S100A12 on the cell death of ANCA-activated neutrophils. **C**–**K** Representative images of the flow cytometry analysis. **C** Neutrophils were incubated with PBS. **D** Neutrophils were incubated with normal-IgG. **E** Neutrophils were incubated with ANCA-IgG alone. **F**–**H** Neutrophils were incubated with ANCA combined with 1, 5, and 10 μg/ml S100A8/A9, respectively. **I**–**K** Neutrophils were incubated with ANCA combined with 1, 5, and 10 μg/ml S100A12, respectively. *p < 0.05, **p < 0.01, ns: not significant

When the concentration of S100A8/A9 in the lower transwell chamber was 5 μg/ml, the number of neutrophils migrating from the upper chamber to the lower chamber reached its maximum value. The chemotaxis index (CI) was 6.263 ± 0.55, which was close to the CI in the positive control group (N-formyl-methionyl-leucyl-phenylalanine, fMLP, 6.503 ± 0.99, *p* = 0.99) (Fig. 4A). S100A12 also had the capability of boosting the migration of neutrophils. When the concentration of S100A12 was 1 μg/ml, the CI was 5.00 ± 0.73 (*p* = 0.08 compared to the fMLP group) (Fig. 4A). To further verify the chemotaxis of S100A8/A9 and S100A12 on ANCA-activated neutrophils, we tested the IL-8 concentration after the stimulation of neutrophils. The results confirmed the elevated IL-8 concentrations after the ANCA-activated neutrophils were treated with S100A8/A9 and S100A12 (Additional file 2: Figure S2A). Treatment with the antibody of TLR4 or RAGE before the addition of S100A8/A9 or S100A12 in the reaction system markedly reduced the IL-8 release from ANCA-activated neutrophils (Additional file 2: Figure S2A).

### S100A8/A9 and S100A12 extend neutrophils lifespan

Isolated neutrophils were treated with ANCA-containing IgG combined with different concentrations of S100A8/A9 or S100A12. The proportion of double negative cells (Annexin V-/PI-) after neutrophils were treated with ANCA-containing IgG was slightly higher than that after neutrophils were treated with normal IgG. However, the difference was not statistically significant (48.57 ± 2.14% vs. 42.97 ± 3.29%, *p* = 0.25). Furthermore, the proportions of Annexin V-/PI- increased after neutrophils were treated with ANCA-containing IgG combined with 1, 5, and 10 μg/ml S100A8/A9 compared with that after neutrophils were treated with ANCA alone (58.37 ± 1.6% and *p* = 0.006, 61.67 ± 4.05% and *p* < 0.001, 57.77 ± 2.6% and *p* = 0.01, respectively). S100A12 also increased the proportions of Annexin V-/PI-. When neutrophils were treated with ANCA-containing IgG plus 1 μg/ml S100A12, the proportion of Annexin V-/PI- increased apparently (compared with neutrophils treated with ANCA alone, 59.70 ± 1.18% vs. 48.57 ± 2.14%, *p* = 0.002) (Fig. 4B–K).

### S100A8/A9 and S100A12 strengthen the ANCA-induced IL-1β release through TLR4 and RAGE

**Fig. 5:**
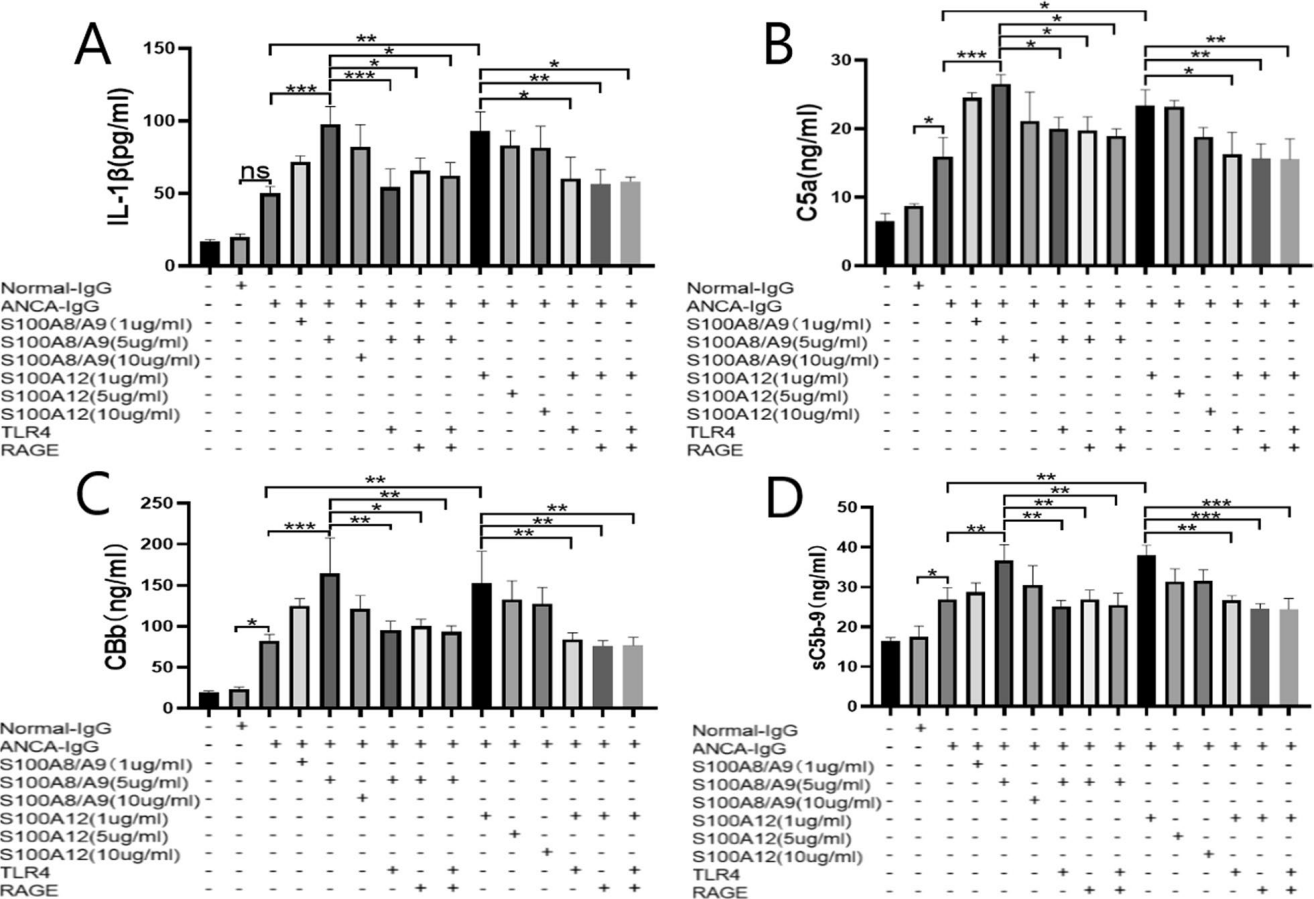
S100A8/A9 and S100A12 enhanced the release of IL-1β and complement activation of ANCA-stimulated neutrophils through TLR4/RAGE. **A** Effects of S100A8/A9 and S100A12 on the release of IL-1β from ANCA-activated neutrophils. **B**–**D** Effects of S100A8/A9 and S100A12 on the supernatant concentrations of C5a, CBb, and sC5b-9 of ANCA-activated neutrophils. *p < 0.05, **p < 0.01, ***p < 0.001, ns: not significant

IL-1β plays an essential role in AAV [13, 14]. Therefore, we investigated whether S100A8/A9 and S100A12 influenced the secretion of IL-1β in ANCA-activated neutrophils. As the results indicated, ANCA-containing IgG stimulated the neutrophils to release more IL-1β in comparison with normal IgG, although without a statistical difference (*p* = 0.056) (Fig. 5A). When neutrophils were incubated with a combination of ANCA and S100A8/A9 or S100A12, the release of IL-1β was further enhanced. The supernatant concentrations of IL-1β decreased obviously when blocking the receptors of TLR4 or RAGE on neutrophils. We also found that the level of IL-1β was further reduced after neutrophils were treated with a combination of TLR4 and RAGE blockade (Fig. 5A).

### S100A8/A9 and S100A12 exaggerate the ANCA-triggered complement activation through TLR4 and RAGE

ANCA-containing IgG could trigger complement activation alone. After neutrophils were incubated with ANCA-containing IgG, increased supernatant C5a (15.93 ± 2.8 ng/ml), CBb (82.60 ± 7.5 ng/ml) and sC5b-9 (26.50 ± 3.01 ng/ml) were detected. When neutrophils were incubated with ANCA plus 5 μg/ml S100A8/A9, the supernatant C5a got to 26.53 ± 1.36 ng/ml (compared with ANCA alone, *p* < 0.001). The combination of ANCA and 1 μg/ml S100A12 also increased supernatant C5a concentration obviously (Compared with ANCA alone, 23.37 ± 2.3 vs. 15.93 ± 2.8 ng/ml, *p* = 0.01). The increased C5a induced by S100A8/A9 or S100A12 was inhibited after neutrophils were pre-incubated with the antibody of TLR4 or RAGE (Fig. 5B).

S100A8/A9 and S100A12 also advanced the ANCA-induced CBb generation. The 5 μg/ml S100A8/A9 has the largest effect (CBb 164.3 ± 43.6 ng/ml, *p* < 0.001, compared with ANCA alone), while the most optimal concentration of S100A12 was 1 μg/ml (CBb 152.4 ± 38.87 ng/ml, *p* = 0.005, compared with ANCA alone). Meanwhile, the effects of S100A8/A9 and S100A12 were also inhibited after the blockade of TLR4 or RAGE (Fig. 5C).

As for the sC5b-9, there was an enhanced generation of sC5b-9 when neutrophils were incubated with ANCA combined with 5 μg/ml S100A8/A9 (36.73 ± 3.95 ng/ml, *p* = 0.008, compared with ANCA alone), while S100A12 with a concentration of 1 μg/ml enhanced the ANCA-induced sC5b-9 generation (38.03 ± 2.48 ng/ml, *p* = 0.002, compared with ANCA alone). Similarly, blocking the receptors of TLR4 and RAGE reduced the effects of S100A8/A9 and S100A12 (Fig. 5D).

To further confirm that S100A8/A9 and S100A12 can promote complement activation in ANCA-activated neutrophils, the mRNA expression of complement C5 was determined by quantitative real-time PCR. The results showed that S100A8/A9 and S100A12 increased complement C5 mRNA expression in ANCA-activated neutrophils. The blockade of TLR4 or RAGE inhibited the effects of S100A8/A9 and S100A12 (Additional file 2: Figure S2B). Western-blot and enzyme-linked immunosorbent assay (ELISA) were also performed to detect the complement C5 protein of PMN lysates after stimulation with S100A8/A9 or S100A12. MPO-ANCA induced the expression of complement C5 in ANCA-activated neutrophils, moreover, S100A8/A9 and S100A12 further promoted the amount of C5 in ANCA-activated neutrophils (Additional file 2: Figures S3A and S4). Additionally, we demonstrated that the expression of complement C5 significantly declined after ANCA-activated neutrophils were pre-treated with the antibody of TLR4 or RAGE (Additional file 2: Figures S3B and S4).

### S100A8/A9 and S100A12 exert pro-inflammatory effects through the p38 MAPK/NF-κBp65 pathway

**Fig. 6:**
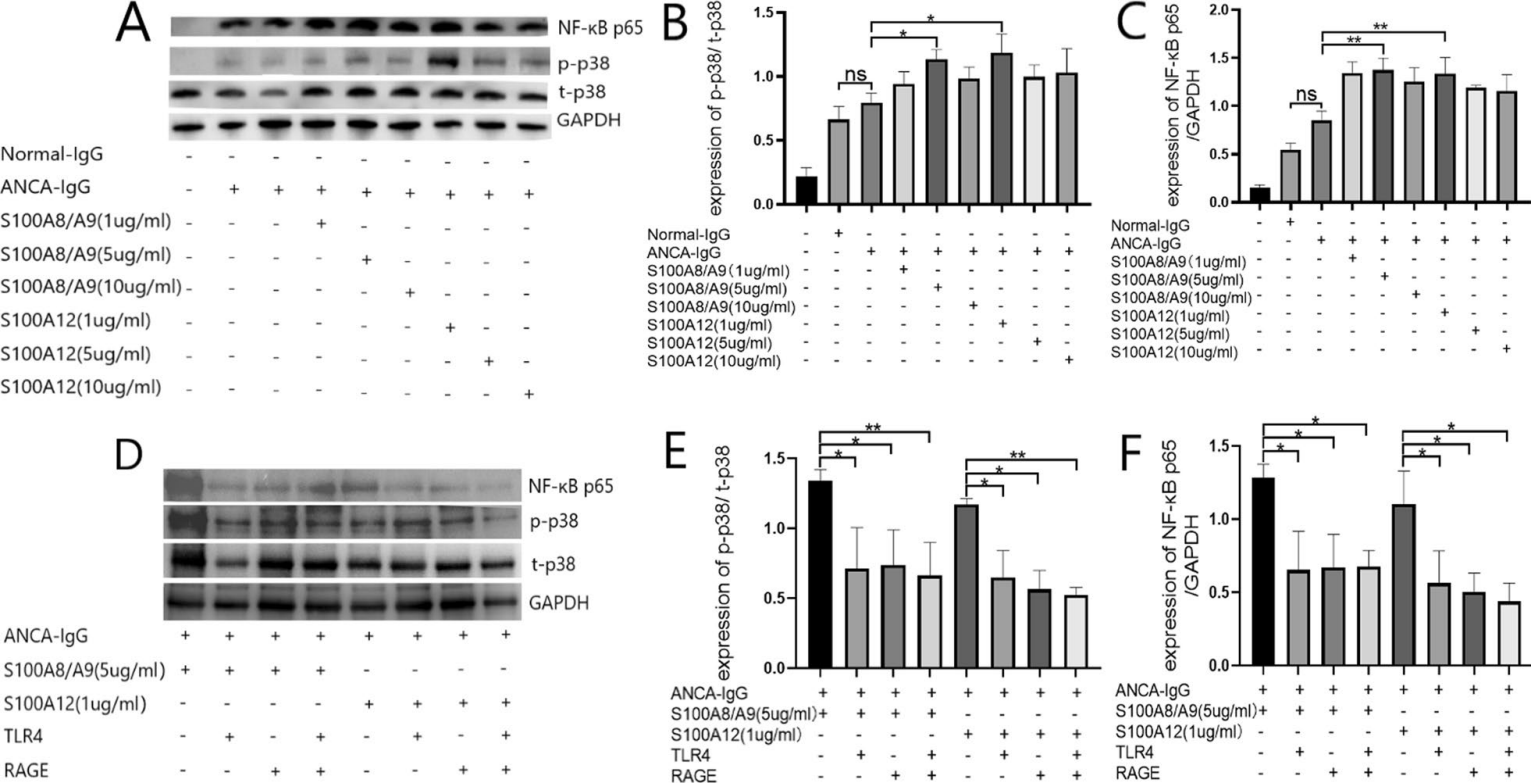
S100A8/A9 and S100A12 played their pro-inflammatory effects through the p38 MAPK/NF-κB p65 pathway. The full-length blot of GAPDH in **A** was absent for the limited exposure space. **A** S100A8/A9 and S100A12 induced p38 MAPK phosphorylation and NF-κB p65 expression. **B** The phosphorylation ratios of p38 after neutrophils were stimulated with a combination of ANCA and S100A8/A9 or S100A12. **C** The activation of NF-κB p65 after neutrophils were stimulated with a combination of ANCA and S100A8/A9 or S100A12. **D** The phosphorylation of p38 MAPK and the activation of NF-κB p65 after the blockade of TLR4 and RAGE. **E** The phosphorylation ratio of p38 after blocking TLR4 and RAGE. **F** The expression of NF-κB p65 after blocking TLR4 and RAGE. *p < 0.05, **p < 0.01, ns: not significant

To further determine whether the effects of S100A8/A9 and S100A12 were dependent on the activation of the MAPK/NF-κB pathway, we analyzed the intracellular expressions of the total p38 MAPK and phosphorylated p38 MAPK, as well as NF-κB p65 using Western-blot. Compared with neutrophils incubated with normal IgG, neutrophils incubated with ANCA-containing IgG showed an elevated phosphorylation ratio of p38 MAPK (phosphorylated p38 MAPK protein/total p38 MAPK), and the expression of NF-κB p65 increased simultaneously. Both S100A8/A9 and S100A12 could enhance the ANCA-induced p38 MAPK phosphorylation and NF-κB p65 expression, which were reduced after the blockade of TLR4, RAGE, and TLR4 + RAGE (Fig. 6). These findings indicated that the activation of the p38 MAPK-NF-κBp65 pathway by S100A8/A9 and S100A12 in AAV was mediated, at least in part, by the TLR4/RAGE.

### The effects of S100A8/A9 and S100A12 do not depend on ROS

It has been reported that S100A8/A9 can inhibit neutrophils’ oxidative metabolism[15]. Compared with normal IgG, ANCA-containing IgG significantly increased the ROS production of neutrophils (MFI, 253,658 ± 9936, *p* < 0.001). When there were S100A8/A9 in the reaction system, the levels of ROS were inhibited. S100A12 did not influence the ROS production of neutrophils induced by ANCA-containing IgG.

**Fig. 7:**
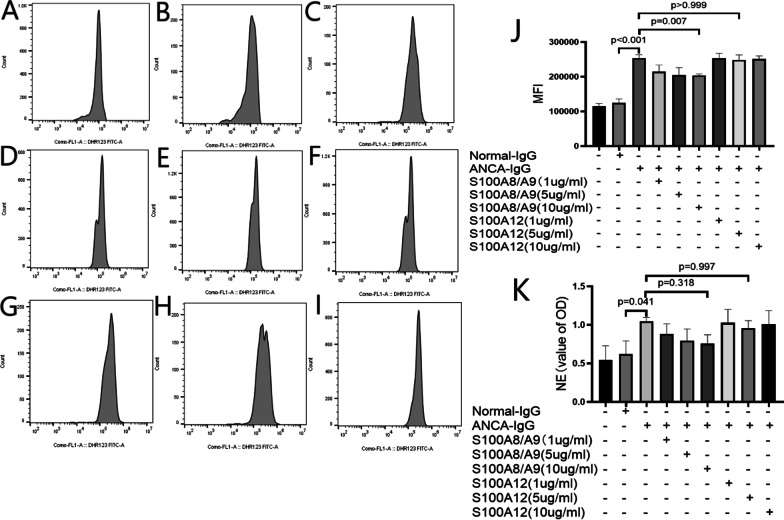
Effects of S100A8/A9 and S100A12 on the production of ROS and NETs in AAV. **A**–**I** Representative images of influences of S100A8/A9 and S100A12 on ANCA-induced production of ROS. Neutrophils were incubated with different stimulators and analyzed using flow cytometry. **A** Neutrophils were incubated with PBS. **B** Neutrophils were incubated with normal-IgG. **C** Neutrophils were incubated with ANCA. **D**–**F** Neutrophils were incubated with ANCA combined with 1, 5, and 10 μg/ml S100A8/A9, respectively. **G**–**I** Neutrophils were incubated with ANCA combined with 1, 5, and 10 μg/ml S100A12, respectively. **J** The mean fluorescence intensity of ROS after neutrophils were incubated with different stimulators. **K** Effects of S100A8/A9 and S100A12 on ANCA-induced formation of NETs. NE: neutrophil elastase

Further, we studied whether S100A8/A9 and S100A12 could influence the ROS-dependent NETs formation [represented by the release of neutrophil elastase (NE)]. As shown in Fig. 7K, S100A8/A9 tended to restrain the release of NE by ANCA-activated neutrophils, and this inhibitory effect reached its maximum value when the concentration of S100A8/A9 got to 10 μg/ml (0.76 ± 0.11, expressed as *A* values at 450 nm). However, there was no statistical difference compared with the release of NE induced by ANCA alone (1.05 ± 0.05, expressed as *A* values at 450 nm and *p* = 0.318). S100A12 did not influence the release of NE induced by ANCA-containing IgG (Fig. 7).

## Discussion

As members of DAMPs, S100A8/A9 and S100A12 play crucial roles in various diseases. Previous studies have reported high levels of S100A8/A9 and S100A12 in AAV patients, and the serum level of S100A8/A9 was related to disease relapse in PR3-AAV [16, 17]. These findings prompted us to clarify the significance of S100A8/A9 and S100A12 in AAV. In the current study, we verified the association between clinical parameters and serum or urine levels of S100A8/A9 and S100A12 in patients with active MPO-AAV. Furthermore, the possible pathogenic roles of S100A8/A9 and S100A12 in MPO-AAV were unveiled with the in vitro experiments.

In the current study, the serum levels of both S100A8/A9 and S100A12 were positively correlated with the serum MPO-ANCA levels. This result differs from the study reported by Pepper RJ [17]. Neutrophils play an important role in the pathophysiology of AAV [18]. It has been demonstrated that S100A8/A9 and S100A12 are primarily released from activated or necrotic neutrophils and are involved in the pathogenesis of various diseases [19]. Since ANCA is an activator of neutrophils, it is reasonable to speculate that the ANCA-activated neutrophils are important sources of the increased serum S100A8/A9 and S100A12 in AAV. We demonstrated that the MPO-ANCA-containing IgG could stimulate neutrophils to release S100A8/A9 and S100A12 dose-dependently.

ANCA-activated neutrophils migrate across endothelial cells and cause inflammation in AAV [20]. Thus, all factors that can enhance the chemotaxis and migration of neutrophils might increase the severity of the disease. Previous studies have reported S100A8/A9 and S100A12 induced neutrophil chemotaxis and adhesion [21, 22]. In the present study, S100A8/A9 and S100A12 dramatically enhanced the chemotaxis of ANCA-activated neutrophils in the transwell experiment. Moreover, we verified that S100A8/A9 and S100A12 promoted the release of IL-8 in neutrophils, which is a potent neutrophil chemotactic factor [23]. This result indicated that in MPO-AAV, S100A8/A9 and S100A12 in the local area of tissue damage would attract more ANCA-activated neutrophils and further amplify the inflammatory response.

Dysregulation of neutrophils’ life span may contribute to the pathogenesis of AAV. Some studies have reported the infiltration and accumulation of unscavenged apoptotic neutrophils in the perivascular tissues and the delayed spontaneous apoptosis of neutrophils in AAV [24, 25]. However, the exact mechanisms have not been clarified. We found that S100A8/A9 and S100A12 prolong survival and decrease the apoptosis of neutrophils. Therefore, S100A8/A9 and S100A12 might contribute to the neutrophil accumulation in regions of inflammation by inhibiting neutrophil apoptosis in AAV.

IL-1β plays a vital role in autoimmune disease as an important pro-inflammatory cytokine. Former literature showed that ANCA could stimulate neutrophils to express mRNA and protein of IL-1β [26]. On the other hand, S100A8/A9 was once reported to stimulate peripheral blood mononuclear cells (PBMCs) to produce IL-1β [27]. Similarly, we found that S100A8/A9 and S100A12 could exaggerate the release of IL-1β through binding to TLR4 and RAGE on neutrophils. This result signified that S100A8/A9 and S100A12 had a pro-inflammatory function in MPO-AAV.

The activation of the alternative pathway of the complement system plays a crucial role in the development of AAV [28, 29]. The raised levels of complement factors in the supernatant and the increased C5 expression of ANCA-activated neutrophils demonstrated that S100A8/A9 and S100A12 could promote the activation of the alternative complement pathway in MPO-ANCA-positive vasculitis. The effects of inhibitors of TLR4 and RAGE further confirmed that the TLR4/RAGE axis is involved in the pathogenic effects of S100A8/A9 and S100A12 in MPO-AAV. It was worth noting that S100A8/A9 and S100A12 also increased the expression of TLR4 and RAGE on ANCA-stimulated neutrophils. This result was consistent with the research of T. H. Page, who demonstrated that the TLR4/RAGE axis is a common pathogenic pathway in AAV [17].

According to previous studies, activations of TLR4 and RAGE can activate the MAPKs and NF-κB, and subsequently enhance the transcription of pro-inflammatory mediators [30, 31]. MAPKs are crucial regulators of a series of cellular processes, such as the proliferation and differentiation of cells [32]. Our data showed that S100A8/A9 and S100A12 increased the expression of phosphorylated p38 MAPK and NF-κB p65, which was reduced by the blockade of TLR4 and RAGE. Therefore, TLR4/RAGE-p38 MAPK-NF-κB p65 signaling pathways were involved in the effects of S100A8/A9 and S100A12 on neutrophils.

A previous report demonstrated that S100A8 reduced reactive oxygen species generated by activated leukocytes through its thiol-scavenging capacity [33]. In the current study, S100A8/A9 inhibited the MPO-ANCA-induced ROS generation of neutrophils. Correspondingly, S100A8/A9 tended to inhibit the ROS-dependent generation of NETs. However, S100A12 did not inhibit the ANCA-induced ROS generation of neutrophils and did not tend to inhibit the generation of NETs.

Some limitations of this study should be mentioned. First, our samples of patients are relatively small, so a larger cohort will be needed to explore the relationship between the level of S100A8/A9 and S100A12 and the prognosis of the AAV. Second, due to the characteristics of AAV in the Chinese population, all patients enrolled in our study were MPO-ANCA positive. Third, all our experiments were done in vitro, so further in vivo study is necessary in the future.

In conclusion, the serum and urine levels of S100A8/A9 and S100A12 in patients with active MPO-ANCA-positive vasculitis were elevated and correlated with the severity of the disease. Besides, S100A8/A9 and S100A12 might take part in the pathogenesis of the disease. Both S100A8/A9 and S100A12 can exaggerate the inflammatory effects of MPO-ANCA in a ROS-independent manner.

## Materials and methods

### Patients

Patients’ serum and urine were obtained from 34 patients with active AAV and positive MPO-ANCA and 8 AAV patients in remission. These 42 patients diagnosed in Tianjin Medical University General Hospital fulfilled the 2012 revised International Chapel Hill Consensus Conference Nomenclature of Vasculitides [2]. Clinical characteristics and laboratory parameters were recorded on the day of sample collection. Disease activity was assessed using version 3 of BVAS. Active patients were defined as those with a BVAS score of more than zero. Patients who got a BVAS score of 0 were identified in remission. Serum and urine from 10 healthy donors were obtained as normal controls. The research complied with the declaration of Helsinki, and the institutional review board of Tianjin Medical University General Hospital approved the protocol (IRB2018-202-01). Informed consent was obtained from all individual participants included in the study.

### Clinical and laboratory data

Clinical data included the following: gender, age, BVAS, disease duration, and organ involvement. Laboratory data included as following: hemoglobin, serum creatinine level, the level of MPO-ANCA, CRP, C3 and C4, ESR, ferritin, D-dimer, RF, albumin, proteinuria, hematuria, urinary NGAL.

### Immunoassays of S100A8/A9 and S100A12

Concentrations of S100A8/A9 (439707, Biolegend) and S100A12 (CSB-E13095h, Cusabio) in serum and urine from participants were measured by ELISA according to the manufacturer’s instructions.

### Neutrophils isolation of peripheral blood

Neutrophils from healthy donors were isolated by density centrifugation [34]. Briefly, a double gradient was formed by layering Histopaque 1077 (10771, Sigma-Aldrich) on an equal volume of Histoque 1119 (11191, Sigma-Aldrich). Heparinized blood donated by healthy volunteers was carefully layered onto the upper gradient. After 20 min centrifugation at 300×*g*, neutrophils between two Histopaque mediums were carefully collected. Cells were washed by adding 10 ml of isotonic phosphate-buffered saline (PBS). The neutrophil pellet was resuspended with red blood cell lysis buffer (R1010, Solarbio) to lyse red blood cells. Neutrophils were then washed twice and resuspended in an appropriate volume of RPMI1640 medium. The concentration of neutrophils was adjusted to 1 × 10^6^/ml.

### Production of S100A8/A9 and S100A12 by neutrophils stimulated with ANCA

Neutrophils (1 × 10^6^/ml) were primed with 2 ng/ml TNF-α (H8916, Sigma-Aldrich) at 37 °C for 15 min and cultured with normal control IgG from healthy donors and different concentrations of ANCA-containing IgG purified from AAV patients at 37 °C for 24 h. The concentrations of S100A8/A9 and S100A12 were detected by ELISA as above.

### TLR4 and RAGE expression on neutrophils

Isolated neutrophils were primed with TNF-α and cultured with various stimulators for 2 h. The expressions of TLR4 and RAGE on neutrophils were performed by flow cytometry. The cells were stained with mouse anti-TLR4 (ab105950, Abcam) and rabbit anti-RAGE (ab228861, Abcam) monoclonal antibodies for 30 min. After washing three times with PBS, neutrophils were stained with PE-conjugated donkey anti-mouse IgG and FITC-conjugated goat anti-rabbit IgG antibodies for 30 min. The cells were resuspended in PBS following washing and detected with the flow cytometer.

### Neutrophil migration and chemotaxis

A sterile 24-well transwell plate with a pore size of 3 μm was used. Cells were pre-stimulated with ANCA-containing IgG (1 mg/ml) for 1 h and were inoculated in the upper chamber. Then the upper chamber was moved to an exploratory well with fMLP (Solarbio), different concentrations of S100A8/A9 (Sino Biological) and S100A12 (Cusabio) in a 37 °C, 5% CO2 cell incubator for 2 h. Five fields of view were randomly selected to take pictures under a 100× optimal microscope. The CI was calculated by dividing the total number of cells in the lower chamber of each experimental group by the total number of cells in the lower chamber of the blank control group. The supernatant concentration of IL-8 was detected by ELISA (ab214030, Abcam).

### Apoptosis of neutrophils

Isolated neutrophils from healthy donors were primed with TNF-α and treated with ANCA, S100A8/A9, or S100A12 for 12 h at a cell incubator. Cells were collected at 1000 rpm and washed twice with PBS. The PMNs pellet was resuspended with binding buffer (10 mM HEPES/NaOH, pH 7.4, 140 mM NaCl, 2.5 mM CaCl_2_). Flow cytometry assessed apoptosis of neutrophils with Annexin V-FITC/PI-PE Kit (FX018, 4A Biotech). The results were analyzed by FlowJo 10.4.0.

### Assessment of IL-1β and complement factors released by neutrophils

TNF-α-primed neutrophils were stimulated for 2 h with normal control IgG, ANCA-containing IgG, S100A8/A9, S100A12, S100A8/A9 + ANCA, S100A12 + ANCA, respectively. In the groups blocking TLR4, RAGE, or TLR4 + RAGE, the antibody of TLR4 (312802, Biolegend) or RAGE (ab37647, Abcam) was added and incubated for 30 min before adding S100A8/A9 and S100A12. The supernatant was collected to detect the concentration of IL-1β (437007, Biolegend), complement 5a (C5a) (JL10644, Shanghai Jianglai Biological Technology Co., Ltd.), complement Bb (CBb) (JL19313, Shanghai Jianglai Biological Technology Co., Ltd.) and soluble complement 5b-9 (sC5b-9) (JL18355, Shanghai Jianglai Biological Technology Co., Ltd.).

### RNA extraction and real-time PCR

Total RNA was extracted from the isolated neutrophils using TRIzol reagent. The quality and integrity of RNA were detected using a NanoDrop ND1000 (Thermo Fisher, USA) and determined via the A260/A280 ratio. Next, the total RNA was reversely transcripted to cDNA using 1st Strand cDNA Synthesis SuperMix (11141ES10, Yeasen) following the manufacturer’s instructions. Real-time quantitative PCR was performed with specific primers by the CFX Manager™ Real-time PCR system (Bio-Rad, USA). Relative changes in mRNA levels were calculated by the 2 − ΔΔCt method. Primer sequences are as follows:

Human *GAPDH*: forward 5′-GGAGCGAGATCCCTCCAAAAT-3′,

reverse 5′-GGCTGTTGTCATACTTCTCATGG-3′;

Human *complement C5*: forward 5′-ACAGTCATAGAGTCTACAGGTGG-3′,

reverse 5′-CCAACTGGTCAAGCGAATCTT-3′.

### Western blot analysis

Neutrophils were collected and added to the RIPA lysis solution and the protease inhibitor PMSF. After 30 min incubation on ice, supernatants were extracted with a 10,000 rpm centrifugation. Denatured PMNs protein extract was subjected to SDS-PAGE and transferred to nitrocellulose membranes, which were then blocked for 1 h at room temperature with 5% skim milk. After incubated with primary antibodies against C5/C5a, p-p38 MAPK/t-p38 MAPK, NF-κB p65 (Abcam, Cambridge, USA), and GAPDH (A19056, ABclonal) overnight at 4 °C, the horseradish peroxidase-conjugated goat anti-mouse or rabbit monoclonal antibody was used to detect the bound primary antibodies. The membranes were exposed with a chemiluminescence imaging system, and the results were performed with the Image J software system (NIH, USA).

### ELISA for the level of C5 protein in PMN lysates

Neutrophils were incubated with different stimulants and collected at 1000 rpm. The lysates of neutrophils were extracted by adding a RIPA lysis solution. The concentration of complement C5 was detected according to the manufacturer’s instruction (ab125963, Abcam).

### Flow cytometry for the oxidative respiratory burst of neutrophils

The measurement of oxidative activation of neutrophils was based on ROS-dependent oxidation of dihydrorhodamine 123 (DHR123) to rhodamine 123 (R123), which is a cationic green fluorescent dye and can derive the uncharged non-fluorescent dye DHR123 [35]. DHR123 was added to the TNF-α primed neutrophils suspension to the final concentration of 5 μg/ml. Neutrophils were then incubated with ANCA-containing IgG (1 mg/ml), normal control IgG (1 mg/ml), S100A8/A9 + ANCA IgG and S100A12 + ANCA IgG at 37 °C for 1 h. The samples were assessed by flow cytometry analysis, and the production of ROS was represented by the mean fluorescence intensity (MFI) of the FITC gating channel.

### Induction of netting neutrophils by S100A8/A9 and S100A12 with ANCA

Neutrophils (1 × 10^6^/ml) were primed with 2 ng/ml TNF-α at 37 °C for 15 min, then incubated with normal control IgG (1 mg/ml), ANCA-containing IgG (1 mg/ml), S100A8/A9 heterodimer protein or S100A12 protein at 37 °C for 24 h. Neutrophils were centrifuged for 5 min at 1500 rpm, and the supernatant was collected. The concentration of NE (JL12352, Shanghai Jianglai Biological Technology Co., Ltd.) was determined by ELISA.

### Statistical analysis

Different experiments were performed at least three times. The normal distribution of quantitative data was tested by the *Kolmogorov–Smirnov* test. Data with normal distribution are expressed as mean ± SD, and median and interquartile ranges are applied for data without normal distribution. Multiple sets of continuous variables which were normally distributed were evaluated using one-way ANOVA analysis followed by Tukey’s test for multiple comparisons. For multiple sets of quantitative data that do not conform to the normal distribution, the Kruskal–Wallis test was used. The Mann–Whitney U test was applied to two-independent groups that were not normally distributed. Categorical variables are presented as frequencies and performed with the χ^2^ test. Correlation analysis was performed by a spearman rank correlation. Statistical significance was set at a *p* value < 0.05. GraphPad Prism 8.0.1 software for Windows (GraphPad Software, California, USA) was used for data analysis.

## Supplementary Information

**Additional file 1**. Full-length images of the cropped blots of Western-blot.

**Additional file 2**. The information of Figures S1 to S5.

## Data Availability

The datasets generated and/or analyzed during the current study are not publicly available due privacy or ethical restrictions but are available from the corresponding author on reasonable request.

## Abbreviations

AAV: ANCA-associated vasculitis
ANCA: Antineutrophil cytoplasmic antibody
MPO: Myeloperoxidase
PR3: Proteinase 3
MPA: Microscopic polyangiitis
GPA: Granulomatosis with polyangiitis
EGPA: Eosinophilic granulomatosis with polyangiitis
PMN: Polymorphonuclear lymphocytes
NETs: Neutrophil extracellular traps
NE: Neutrophil elastase
DAMP: Damage-associated molecular pattern
HMGB1: High mobility group box protein 1
BVAS: Birmingham vasculitis activity score
CRP: C reactive protein
ESR: Erythrocyte sedimentation rate
RF: Rheumatoid factor
NGAL: Neutrophil gelatinase-associated lipocalin
PBS: Phosphate-buffered saline
CI: Chemotaxis index
fMLP: *N*-Formyl-methionyl-leucyl-phenylalanine
TLR4: Toll-like receptor 4
RAGE: Receptor of advanced glycation endproducts
PMSF: Phenylmethylsulfonyl fluoride
MAPK: Mitogen-activated protein kinase
NF-κB: Nuclear factor-kappa B
GAPDH: Glyceraldehyde-3-phosphate dehydrogenase
ROS: Reactive oxygen species
MFI: Mean fluorescence intensity
PBMC: Peripheral blood mononuclear cells
