# Supplementary material for: The potential pathogenic roles of S100A8/A9 and S100A12 in patients with MPO-ANCA-positive vasculitis
Source: BMC Immunol. 2022 Sep 10;23:42. doi: 10.1186/s12865-022-00513-4 (PMC9464401; doi:10.1186/s12865-022-00513-4)
Supplement: Supplementary file 1 — Additional file 1. Full-length images of the cropped blots of Western-blot. [file 12865_2022_513_MOESM1_ESM.pdf]

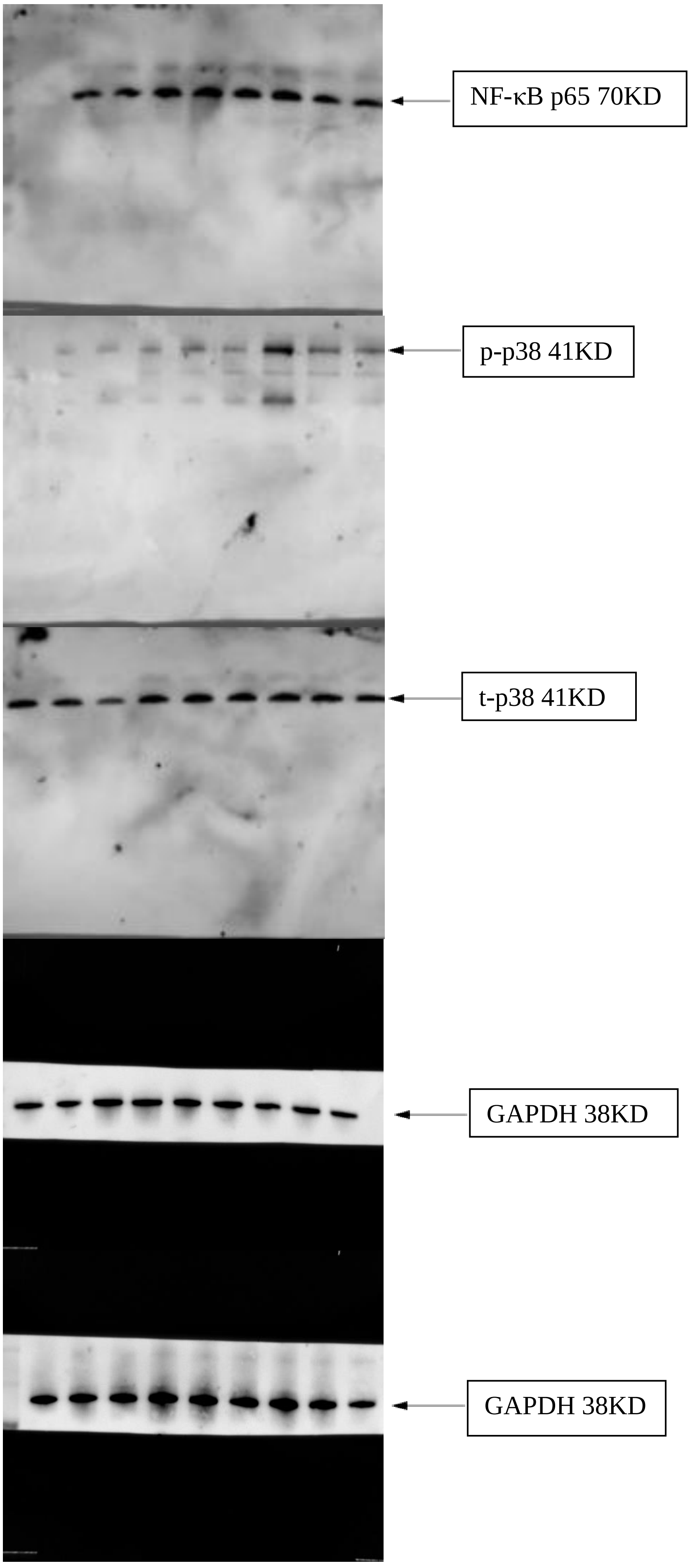

NF-κB p65 70KD
p-p38 41KD
t-p38 41KD
GAPDH 38KD
GAPDH 38KD

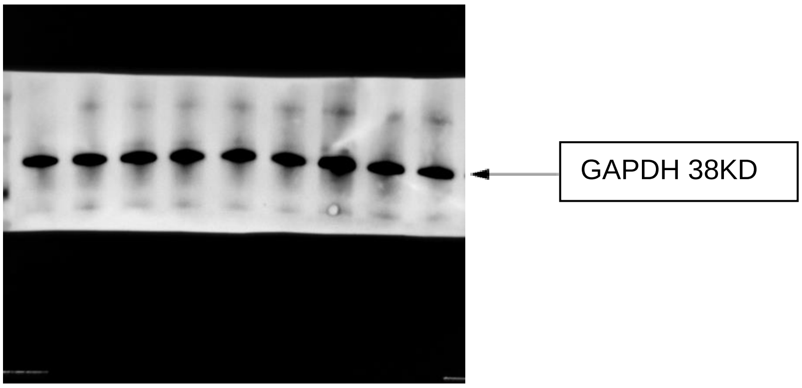

Supplementary material 1: The full-length blots above are supplements of Figure 6A. Because of the limited space, the membrane edges on both sides cannot be saved, or the full-length blots would be missed. To demonstrate the authenticity of the first GAPDH blot, which is the original images of the cropped blots in Figure 6A, we added two other replicates of GAPDH. The lanes from left to right are the groups of blank, normal-IgG, ANCA-IgG, ANCA+S100A8/A9(1ug/ml), ANCA+S100A8/A9(5ug/ml), ANCA+S10 0A8/A9(10ug/ml), ANCA+S100A12(1ug/ml), ANCA+S100A12(5ug/ml), ANCA+S1 00A12(10ug/ml) respectively.

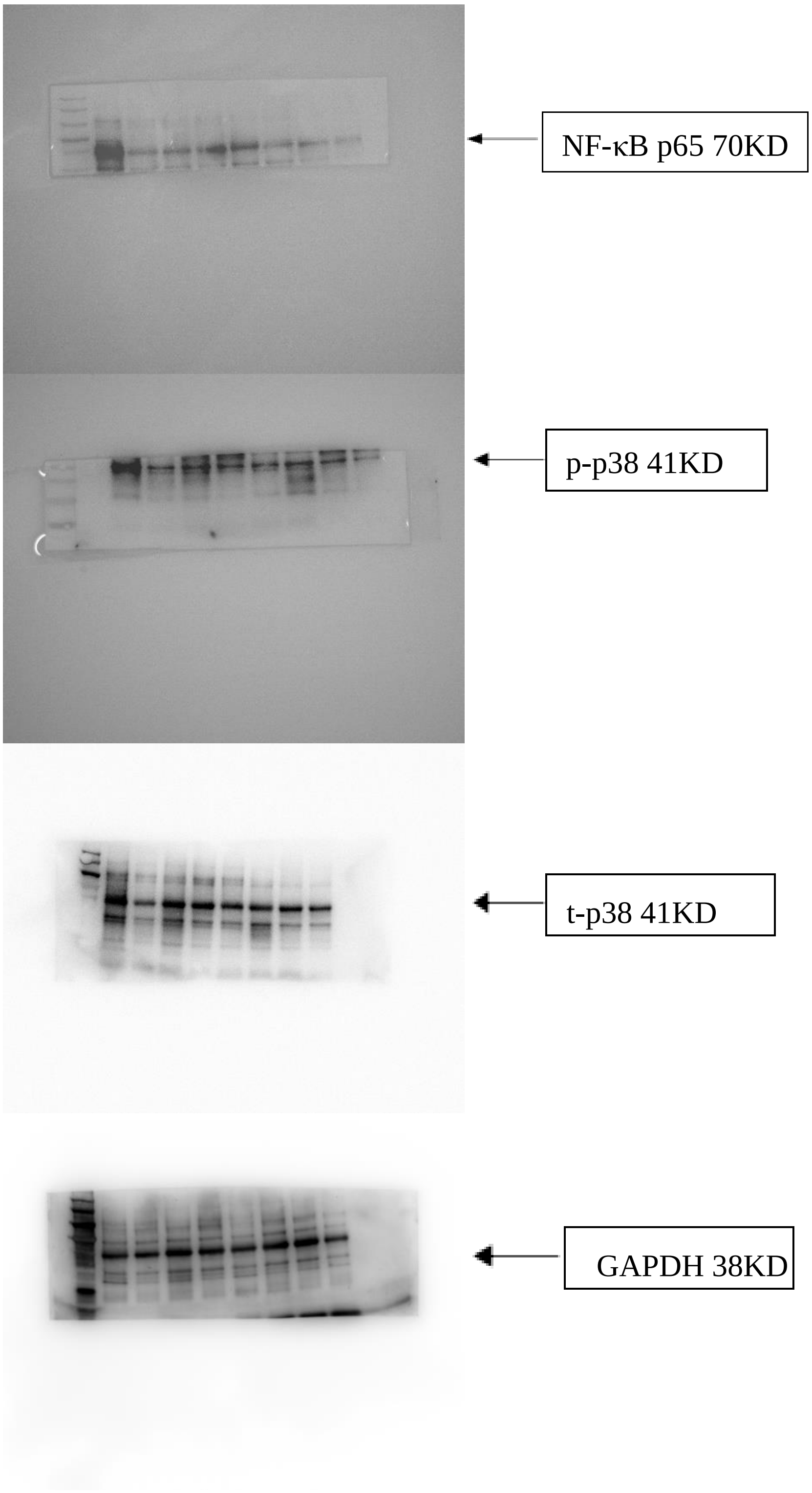

Supplementary material 2: The full-length blots above are supplements of Figure 6D. The last eight lanes from left to right are the groups of ANCA+S100A8/A9(5ug/ml), ANCA+S100A8/A9(5ug/ml)+TLR4, ANCA+S100A8/A9(5ug/ml)+RAGE, ANCA+S100A8/A9(5ug/ml)+TLR4+RAGE, ANCA+S100A12(1ug/ml), ANCA+S100A12(1ug/ml)+TLR4, ANCA+S100A12(1ug/ml)+RAGE, ANCA+S100A12(1ug/ml)+TLR4+RAGE respectively.

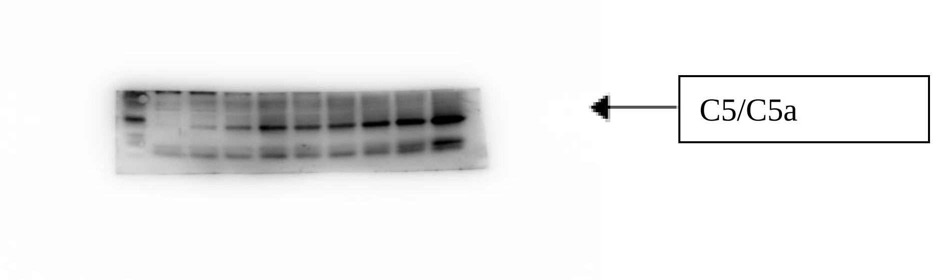

C5/C5a

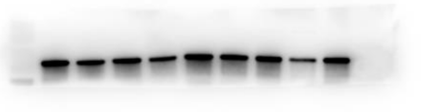

GAPDH

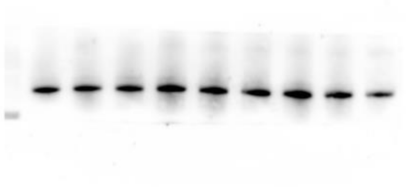

GAPDH

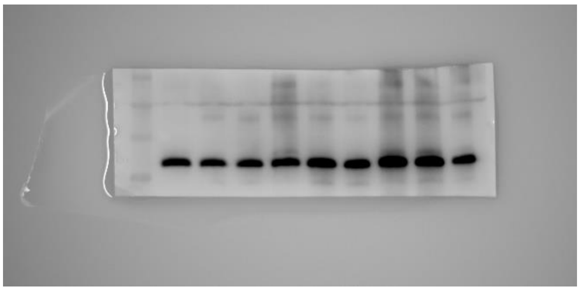

GAPDH

Supplementary material 3：The full-length blots above are supplements of Figure S3A. To demonstrate the authenticity of the first GAPDH blot, which is the original images of the cropped blots in Figure S3A, we added two other replicates of GAPDH.The lanes from left to right are the groups of blank, normal-IgG, ANCA-IgG, ANCA+S100A8/A9(1ug/ml), ANCA+S100A8/A9(5ug/ml), ANCA+S10 0A8/A9(10ug/ml), ANCA+S100A12(1ug/ml), ANCA+S100A12(5ug/ml), ANCA+S1 00A12(10ug/ml) respectively.

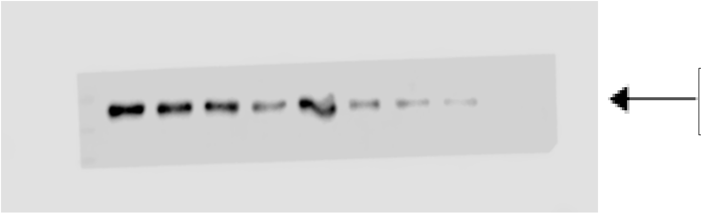

C5/C5a

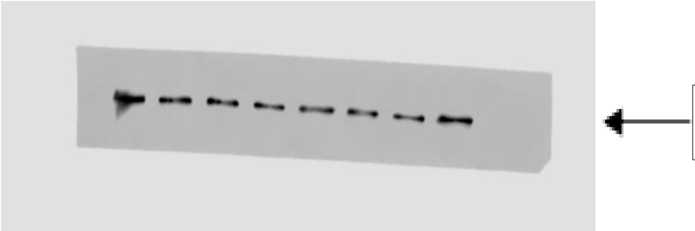

GAPDH

Supplementary material 4: The full-length blots above are supplements of Figure S3B.

The lanes from left to right are the groups ANCA+S100A8/A9(5ug/ml), ANCA+S100A8/A9(5ug/ml)+TLR4, ANCA+S100A8/A9(5ug/ml)+RAGE, ANCA+S100A8/A9(5ug/ml)+TLR4+RAGE, ANCA+S100A12(1ug/ml), ANCA+S100A12(1ug/ml)+TLR4, ANCA+S100A12(1ug/ml)+RAGE, ANCA+S100A12(1ug/ml)+TLR4+RAGE respectively.

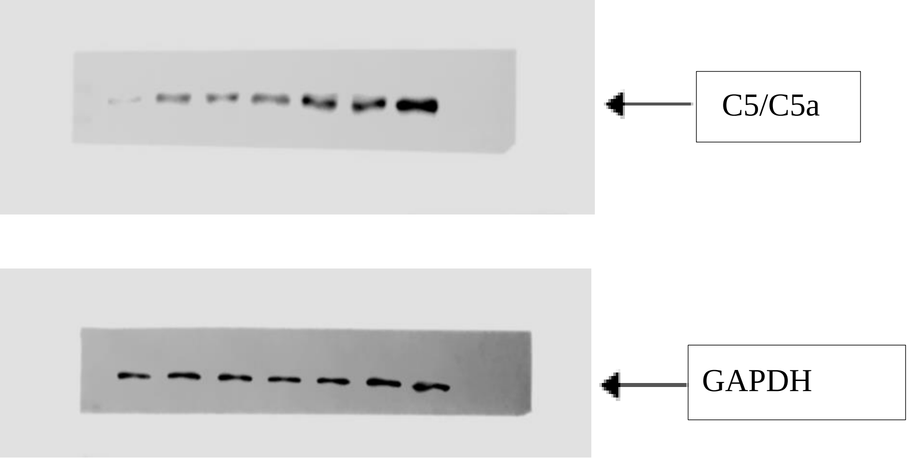

Supplementary material 5: The full-length blots above are supplements of Figure S5E. The lanes from left ro right are the groups of blank, normal-IgG (150ug/ml), normal-IgG (500ug/ml), normal-IgG (1mg/ml), ANCA-IgG (150ug/ml), ANCA-IgG (500ug/ml), ANCA-IgG (1mg/ml) respectively.
