# Supplementary material for: The potential pathogenic roles of S100A8/A9 and S100A12 in patients with MPO-ANCA-positive vasculitis
Source: BMC Immunol. 2022 Sep 10;23:42. doi: 10.1186/s12865-022-00513-4 (PMC9464401; doi:10.1186/s12865-022-00513-4)
Supplement: Supplementary file 2 — Additional file 2. The information of Figures S1 to S5. [file 12865_2022_513_MOESM2_ESM.pdf]

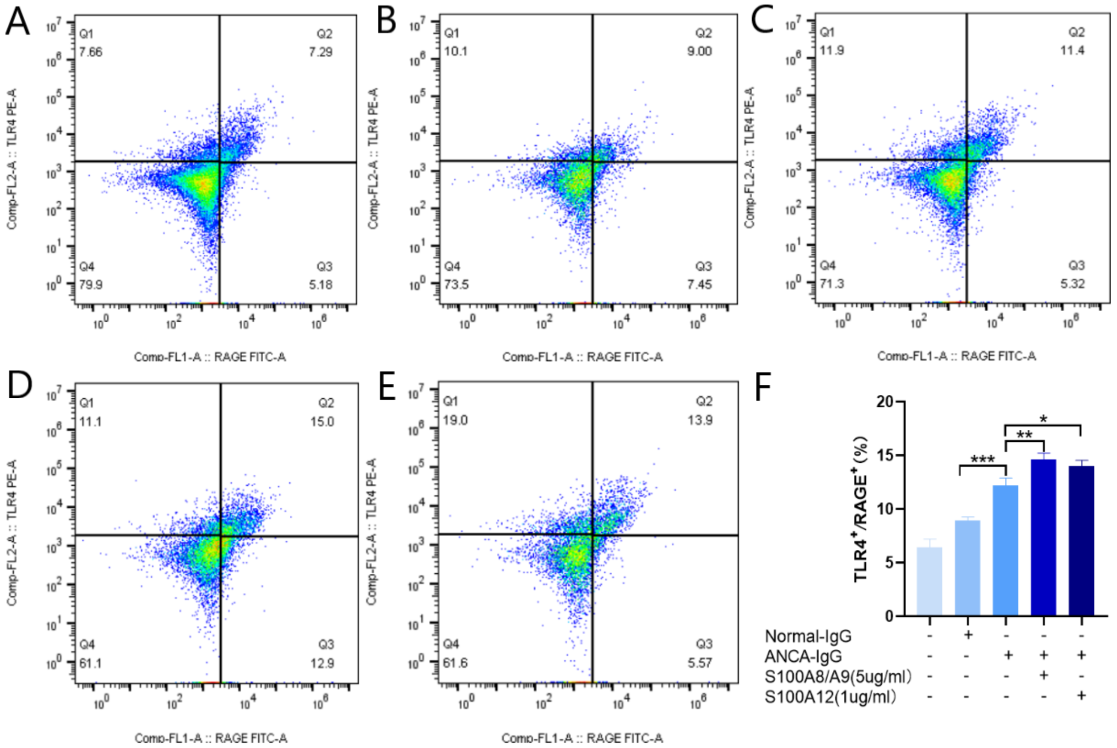

Figure S1. A-E: The expression of TLR4 and RAGE on neutrophils treated with PBS (A), normal IgG (B), ANCA-IgG (C), ANCA+S100A8/A9 (5μg/ml) (D), and ANCA+S100A12 (1μg/ml) (E), respectively. F: The proportion of $TLR4^{+}$ $RAGE^{+}$ neutrophils. *P<0.05, **P<0.01,***P<0.001.

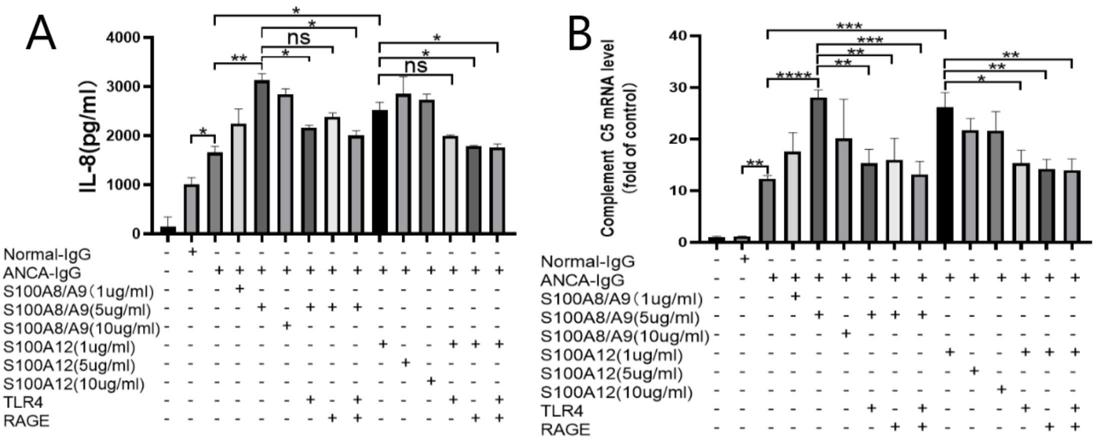

Figure S2. A: Levels of IL-8 in the supernatant of different groups were detected by ELISA. B: The expression of C5 mRNA in neutrophils of different groups was determined by quantitative real-time PCR. *P<0.05, **P<0.01,***P<0.001, ****P<0.0001, ns: not significant.

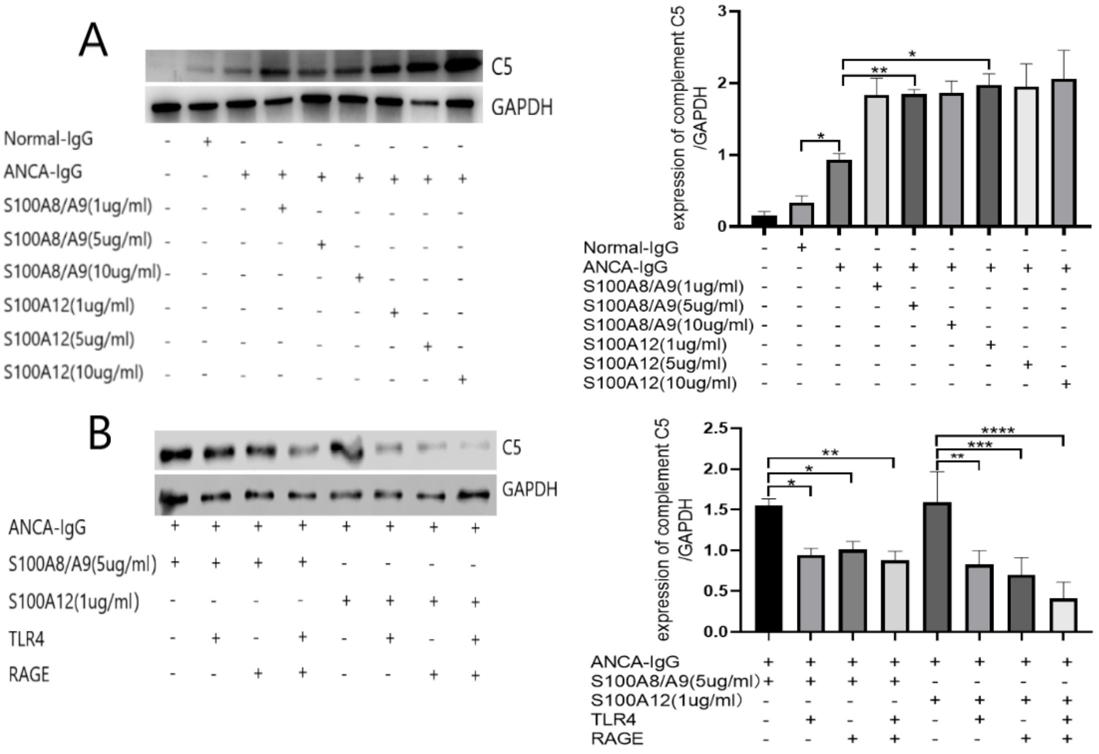

Figure S3. S100A8/A9 and S100A12 promoted the expression of complement C5 protein in ANCA-induced neutrophils through TLR4/RAGE. The full-length blot of GAPDH in figure S3A was vague for the strong exposure intensity. A: The C5 protein expression of neutrophils after incubated with different stimulants. B: Western-blot analysis of complement C5 expression in PMNs after the blockade of TLR4 and RAGE. *P<0.05, **P<0.01,***P<0.001, ****P<0.0001.

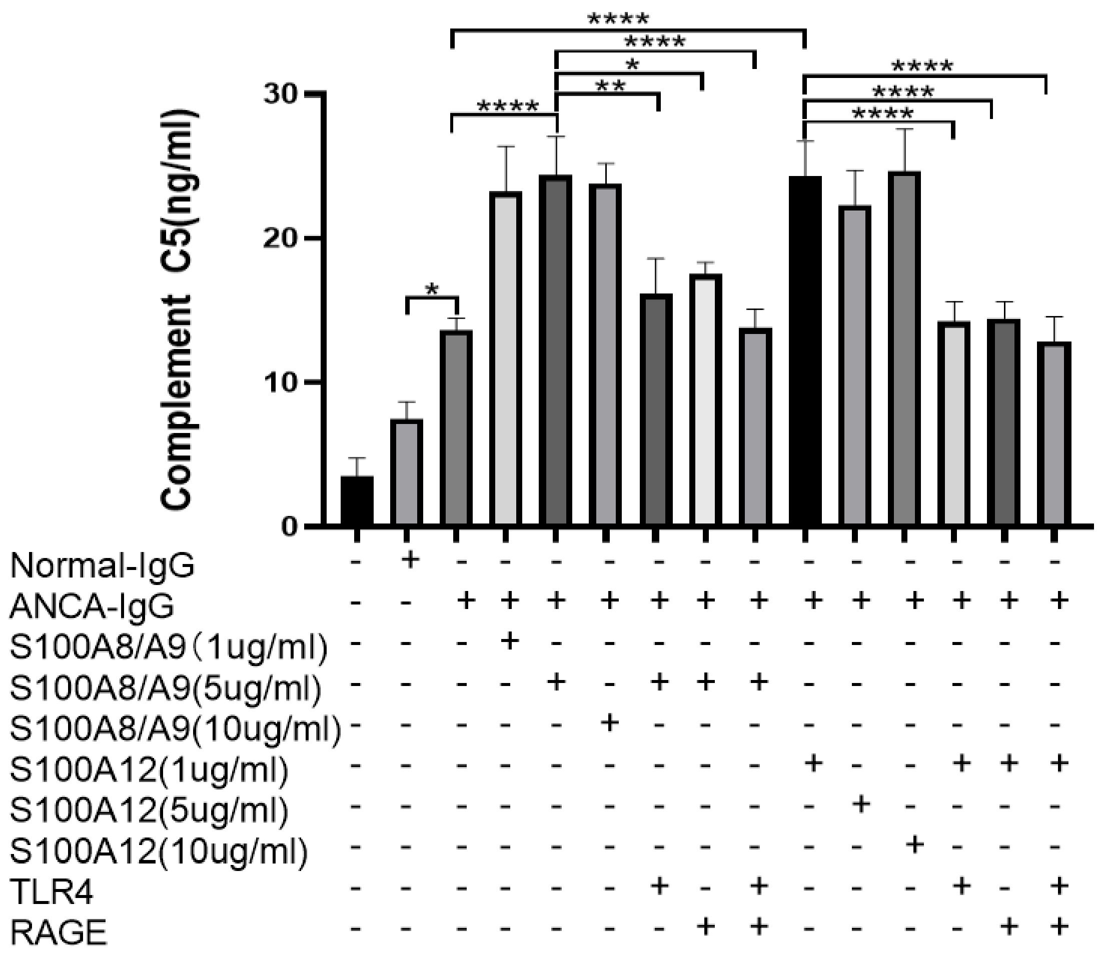

Figure S4. ELISA of complement C5 of PMN lysates. *P<0.05, **P<0.01,***P<0.001, ****P<0.0001.

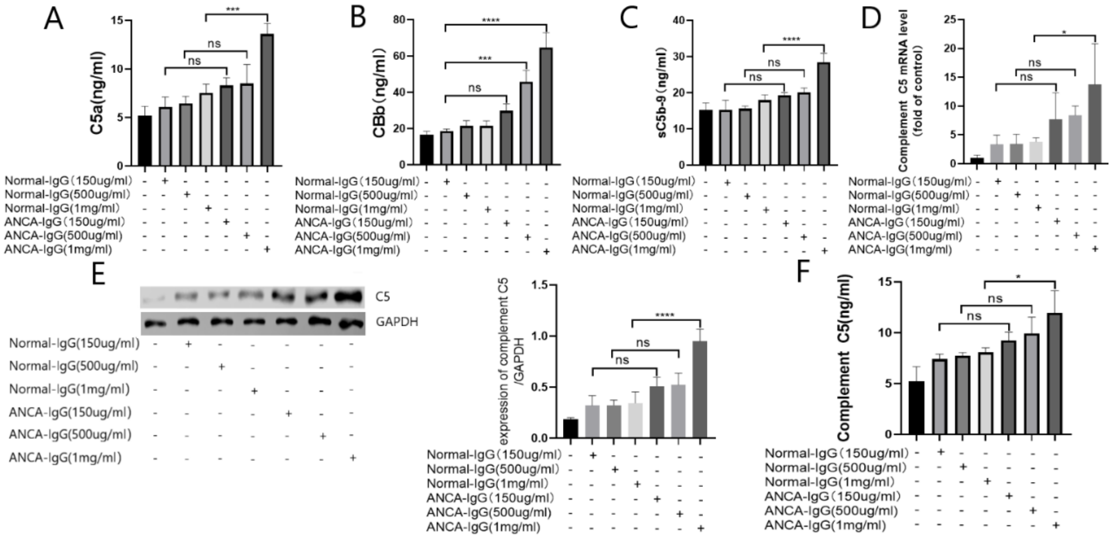

Figure S5. Lower ANCA IgG concentrations were tested for the release of complement factors. The level of C5a (A), CBb (B), and sC5b-9 (C) in the groups of lower ANCA IgG concentrations and their matched control IgG. D: The expression of C5 mRNA in the groups of lower ANCA IgG concentrations. E: Western-blot analysis of complement C5 protein in the groups of lower ANCA IgG concentrations. F: Complement C5 protein of PMN lysates detected by ELISA. *P<0.05, **P<0.01,***P<0.001, ****P<0.0001, ns: not significant.
